# Chronic Fatigue and Dysautonomia following COVID-19 Vaccination Is Distinguished from Normal Vaccination Response by Altered Blood Markers

**DOI:** 10.3390/vaccines11111642

**Published:** 2023-10-26

**Authors:** Amelie Semmler, Anna Katharina Mundorf, Anna Sabrina Kuechler, Karin Schulze-Bosse, Harald Heidecke, Kai Schulze-Forster, Matthias Schott, Markus Uhrberg, Sandra Weinhold, Karl J. Lackner, Marc Pawlitzki, Sven Guenther Meuth, Fritz Boege, Jana Ruhrländer

**Affiliations:** 1 Central Institute for Clinical Chemistry and Laboratory Diagnostics, Medical Faculty, University Hospital Düsseldorf, Heinrich-Heine-University, 40225 Düsseldorf, Germany; amsem100@uni-duesseldorf.de (A.S.); annakatharina.mundorf@med.uni-duesseldorf.de (A.K.M.); anna.kuechler@med.uni-duesseldorf.de (A.S.K.); karin.schulze-bosse@med.uni-duesseldorf.de (K.S.-B.); 2 Cell Trend GmbH, 14943 Luckenwalde, Germany; heidecke@celltrend.de (H.H.); schufo@celltrend.de (K.S.-F.); 3 Division for Specific Endocrinology, Medical Faculty, University Hospital Düsseldorf, Heinrich-Heine-University, 40225 Düsseldorf, Germany; matthias.schott@med.uni-duesseldorf.de; 4 Institute for Transplantation Diagnostics and Cell Therapeutics, Medical Faculty, University Hospital Düsseldorf, Heinrich-Heine-University, 40225 Düsseldorf, Germany; markus.uhrberg@med.uni-duesseldorf.de (M.U.); sandra.weinhold@med.uni-duesseldorf.de (S.W.); 5 University Medical Center, Johannes Gutenberg-University Mainz, 55101 Mainz, Germany; karl.lackner@unimedizin-mainz.de; 6 Department of Neurology, Heinrich-Heine-University, 40225 Düsseldorf, Germany; marcguenter.pawlitzki@med.uni-duesseldorf.de (M.P.); svenguenther.meuth@med.uni-duesseldorf.de (S.G.M.); 7 Selbsthilfegruppe Post-Vac-Syndrom Deutschland e.V., 34121 Kassel, Germany; jana.ruhrlaender@gmx.de

**Keywords:** post-acute COVID-19 vaccination syndrome, PACVS, G-protein-coupled receptor, receptor antibody, interleukin-6, dysautonomia, chronic fatigue

## Abstract

SARS-CoV-2 mRNA vaccination can entail chronic fatigue/dysautonomia tentatively termed post-acute COVID-19 vaccination syndrome (PACVS). We explored receptor autoantibodies and interleukin-6 (IL-6) as somatic correlates of PACVS. Blood markers determined before and six months after first-time SARS-CoV-2 vaccination of healthy controls (*N* = 89; 71 females; mean/median age: 39/49 years) were compared with corresponding values of PACVS-affected persons (*N* = 191; 159 females; mean/median age: 40/39 years) exhibiting chronic fatigue/dysautonomia (≥three symptoms for ≥five months after the last SARS-CoV-2 mRNA vaccination) not due to SARS-CoV-2 infection and/or confounding diseases/medications. Normal vaccination response encompassed decreases in 11 receptor antibodies (by 25–50%, *p* < 0.0001), increases in two receptor antibodies (by 15–25%, *p* < 0.0001) and normal IL-6. In PACVS, serological vaccination–response appeared significantly (*p* < 0.0001) altered, allowing discrimination from normal post-vaccination state (sensitivity = 90%, *p* < 0.0001) by increased Angiotensin II type 1 receptor antibodies (cut-off ≤ 10.7 U/mL, ROC-AUC = 0.824 ± 0.027), decreased alpha-2B adrenergic receptor antibodies (cut-off ≥ 25.2 U/mL, ROC-AUC = 0.828 ± 0.025) and increased IL-6 (cut-off ≤ 2.3 pg/mL, ROC-AUC = 0.850 ± 0.022). PACVS is thus indicated as a somatic syndrome delineated/detectable by diagnostic blood markers.

## 1. Introduction

The onset of chronic, debilitating symptoms following SARS-CoV-2 vaccination is thought to constitute a novel disease entity, for which the term post-acute COVID-19 vaccination syndrome (PACVS) has recently been suggested [1]. The symptoms reported by PACVS-affected persons start shortly after SARS-CoV-2 vaccination, continue in episodes over several months, and severely compromise the quality of life. A systematic survey of the clinical features of PACVS has yet to be carried out. However, published case reports [1] indicate that PACVS differs from the usual adverse effects of SARS-CoV-2 vaccination [2,3,4,5]. The symptoms most frequently reported in the context of PACVS encompass impaired well-being (exhaustion, malaise, chronic fatigue), cardiovascular disturbances (orthostatic intolerance, tachycardia, palpitations), peripheral neuropathy (dysesthesia, hypesthesia), central nervous system dysfunction (lack of concentration, brain fog, cognitive deficits, sleep disorders), muscular dysfunction (myalgia, weakness, fibrillations), and gastro-intestinal afflictions (nausea, strong weight changes). In summary, PACVS presents a phenotype of acquired autonomous dysfunction that overlaps with various established multisystemic dysautonomia syndromes such as myalgic encephalomyelitis/chronic fatigue syndrome (ME/CFS) [6,7], postural orthostatic tachycardia syndrome (POTS) [8], fibromyalgia/chronic pain syndrome [9], small fiber neuropathy (SFN) [10] and mast cell activation syndrome (MCAS) [11]. Interestingly, symptoms similarly conforming to ME/CFS and POTS have been observed following vaccinations against human papillomavirus [12,13,14,15,16] and hepatitis B virus [17].

Dysautonomia syndromes unrelated to vaccination are frequently associated with alterations of humoral autoimmunity against receptors and transmitters involved in autonomous regulation [18,19,20,21]. Increases in circulating levels of these antibodies are linked to the incidence, duration and severity of ME/CFS [22] and POTS [23,24,25]. IgG-directed therapy has been successful in ameliorating symptoms [26,27]. Increases in circulating receptor antibodies were also observed in severe COVID-19 [28,29,30,31,32], which similarly exhibits ME/CFS-like symptoms [33] amenable to IgG-directed therapy [34].

Taken together, the above considerations prompt the hypothesis that antibodies against autonomous regulation elements could play a role in PACVS and possibly serve as therapeutic targets or diagnostic markers. To address this hypothesis, we have here investigated the impact of SARS-CoV-2 vaccination on receptor antibodies known to be involved in POTS [20,23,24], ME/CFS [18,22,25] and immune homeostasis [35]. Circulating levels of these antibodies were measured before and six months after vaccination in normal healthy individuals not affected by PACVS. Normal post-vaccination levels were compared with corresponding levels of a matched cohort presumed to be affected by PACVS because exhibiting persistent symptoms of chronic severe autonomous dysfunction [6,7,8,9,10,11] following SARS-CoV-2 vaccination.

## 2. Materials and Methods

### 2.1. Study Participants

Study participants exhibiting PACVS following SARS-CoV-2 vaccination (*N* = 191, *N =* 32 males, mean/median age = 40/39 years) were recruited from self-help groups using online questionnaires. Participants were diagnosed with ME/CFS, POTS, or related/overlapping syndromes (fibromyalgia/chronic pain syndrome, SFN and MCAS) and/or exhibited at least three symptoms conforming to these syndromes [6,7,8,9,10,11] (details in Table S2). A comparable list of symptoms has recently been observed in chronic sequelae of COVID-19 [36]. Participants were only included if the above diagnoses or symptoms were confirmed by a physician/in a hospital and had persisted for five months or more following vaccination. The vaccination regimen preceding PACVS encompassed one (47 cases), two (96 cases) or three cycles (48 cases) of vaccination with Spikevax, Moderna (32 cases) or Comirnaty, Pfizer/BioNTech (159 cases). In 17 cases, the mRNA vaccination causing PACVS was preceded by one vaccination cycle with a vector-based vaccine (details in Table S1). Exclusion criteria encompassed (i) occurrence of the above symptoms after other vaccinations (including non-mRNA SARS-CoV-2 vaccinations) and/or after acute SARS-CoV-2 infection, (ii) pre-vaccination histories of ME/CFS, POTS or other potentially confounding diseases or syndromes, (iii) confounding pre-medications (details in Table S2). Of 1500 individuals applying for study participation, 1309 were excluded (Figure S3).

### 2.2. Controls

Healthy controls (*N* = 89, *N* = 18 males, mean/median age = 39/49 years) matched for gender and chronological age (*p* < 0.001, U-test) were recruited from a surveillance study of healthy hospital employees subjected to initial dual vaccination with SARS-CoV-2 mRNA vaccine (Spikevax, Moderna) [37]. Paired serum samples were obtained 48 h before the first vaccination and six months after the second vaccination. Control candidates were excluded when they reported disease symptoms or exhibited serological evidence of inflammation, cardiac dysfunction or inter-current SARS-CoV-2 infection during the surveillance period of six months after the second vaccination (details in Table S2, Figure S3).

### 2.3. Validation of SARS-CoV-2 Vaccination and Infection

SARS-CoV-2 vaccination response in controls and PACVS-affected study subjects was confirmed in all cases by sero-reactivity against SARS-CoV-2 spike S1 protein (SAB). Completed SARS-CoV-2 infections were identified by sero-reactivity against SARS-CoV-2 nucleocapsid protein (NAB) [37]. Controls were excluded if they were NAB-reactive or reported SARS-CoV-2 infections and/or COVID-19 re-convalescence in their case history. Study participants were excluded if suffering from florid (PCR-positive) SARS-CoV-2 infection.

### 2.4. Ethics

Clinical trial protocols were approved by the local ethics board of Heinrich-Heine University Düsseldorf (study numbers 2022-1948 and 2020-1259). The investigation conforms with the principles outlined in the World’s Medical Association Declaration of Helsinki. Before inclusion in the study, all participants provided written informed consent.

### 2.5. Laboratory Measurements

Serum was collected by antecubital vein puncture, processed by accredited procedures and stored for up to 6 months at −20 °C. Antibodies against AT1R (Angiotensin II type 1 receptor), ETAR (Endothelin-1 type A receptor), IL-1-Rb (Interleukin-1 receptor type 2), α1-adr-R (Alpha-1 adrenergic receptor), α2a-adr-R (Alpha-2A adrenergic receptor), α2b-adr-R (Alpha-2B adrenergic receptor), α2c-adr-R (Alpha-2C adrenergic receptor), β1-adr-R (Beta-1 adrenergic receptor), β2-adr-R (Beta-2 adrenergic receptor), M1R–M5R (muscarinic acetylcholine receptor M1–M5), MASR (MAS 1 receptor) and ACE-II (Angiotensin-converting enzyme 2) were measured in sera using commercially available immuno-assays (CellTrend GmbH, Luckenwalde, Germany) according to the instructions of the manufacturer. Briefly, we determined IgG-binding to microtiter plates coated with native plasma membranes of cells overexpressing the respective receptors. Bound IgG was detected by secondary antibodies and the biotin/streptavidin system. Each serum sample was determined in duplicate. Assays were calibrated with polyclonal standard sera, yielding quantitative values for receptor-specific IgG expressed in arbitrary units/mL. PanIg antibodies against SARS-CoV-2 spike S1 protein (SAB) and nucleocapsid protein (NAB) were determined as previously described [37]. All other laboratory tests including for interleukin 6 (IL-6), interleukin 8 (IL-8) and C-reactive protein (CRP) were performed following accredited routine laboratory diagnostic procedures. Unless stated otherwise, reference values followed the recommendations of the International Federation of Clinical Chemistry (IFCC).

### 2.6. Statistical Methods

Graph Pad Prism 9 (Graph Pad Software, Inc., San Diego, CA, USA, Graph Pad Prism 9 for Apple Macintosh, released 2020) was used for analysis. Normal distribution was tested using the Shapiro–Wilk test. Non-normally distributed data was presented in the form of mean/median values and interquartile ranges. Differences between controls before and after vaccination were analyzed using the *t*-test for paired samples (two-tailed). Differences between study subjects and controls were analyzed using the Mann–Whitney U test (two-tailed). Correlations were assumed to be good at Spearman’s R ≥ 0.7. For all tests, statistical significance was assumed at *p* < 0.0001. Missing data were managed by listwise deletion.

## 3. Results

### 3.1. Impact of SARS-CoV-2 Vaccination on Receptor Antibodies in Healthy Controls

**Figure 1:**
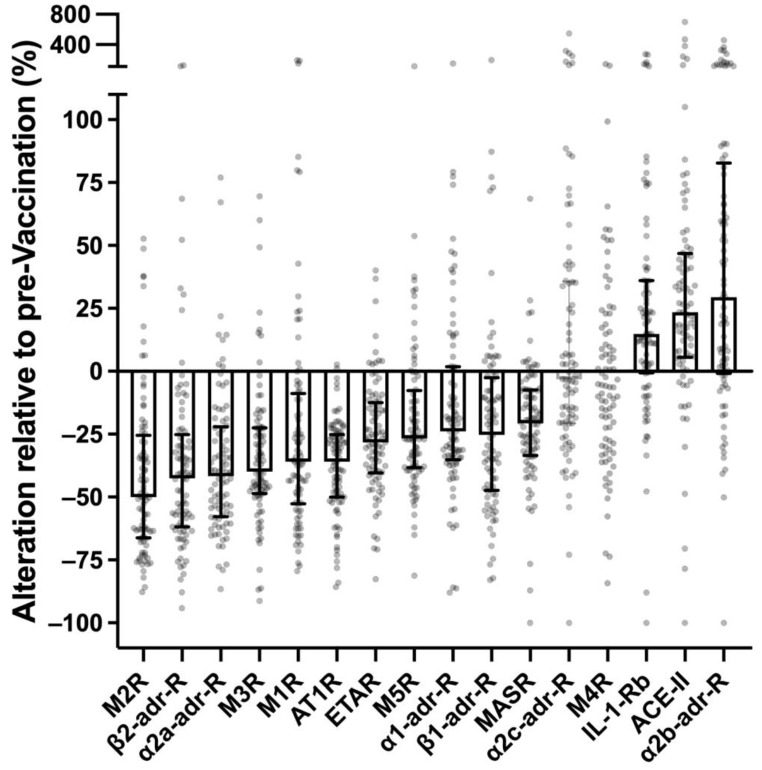
Impact of SARS-CoV-2 mRNA vaccination on receptor antibodies in healthy controls. Levels of GPCR antibodies in the sera of healthy volunteers (N = 89) six months after 2nd vaccination with Spikevax (Moderna) expressed as % of corresponding values obtained immediately before 1st vaccination. Boxes and error bars: medians ± interquartile ranges of significant changes (paired *t*-test, *p* < 0.0001).

Control sera were collected during the first vaccination with two cycles of the mRNA vaccine Spikevax (Moderna). Samples were obtained 48 h before the first vaccination and six months after the second vaccination from 89 healthy individuals not reporting adverse vaccination reactions persisting for more than two weeks after complete vaccination and not suffering from potentially confounding diseases. In normal pairs of vaccination-naïve and post-vaccination sera, the circulating levels of IgG specific for various receptors (expressed as U/mL) were to some extent covariant with each other. Circulating levels of receptor antibodies were not correlated or co-variant, either before or after vaccination, with chronological age, gender, total IgG, brain natriuretic pro-peptide (pBNP) or interleukin 6 (IL-6), thus excluding these factors as potential analytic confounders (Figure S1). Almost all potential disease-relevant receptor antibodies differed markedly between pre- and post-vaccination sera (Figure 1). In post-vaccination sera, the levels of antibodies against AT1R, ETAR, M1R, M2R, M3R, M5R, α1-adr-R, α2a-adr-R, β1-adr-R, β2-adr-R and MASR were markedly lower (in median by 25–50%), while the levels of antibodies against IL-1-Rb, ACE-II and α2b-adr-R were markedly higher (in median by 15–25%). Only two of the analyzed receptor antibodies (α2c-adr-R and M4R) were unaffected by vaccination. Vaccination responses of circulating receptor antibodies were highly significant (*p* < 0.0001, paired t-test) and persisted for at least six months after the last vaccination shot. It should be emphasized that the marked impact of SARS-CoV-2 vaccination on circulating levels of certain receptor antibodies was observed in healthy individuals not exhibiting any long-term disease symptoms following vaccination. These alterations can therefore be considered a normal (non-pathological and non-pathognomonic) reaction or adaptation of humoral receptor autoimmunity to vaccinations with SARS-CoV-2 mRNA vaccines.

### 3.2. GPCR Antibodies in Post-Vaccination Controls and PACVS-Affected Subjects

**Figure 2:**
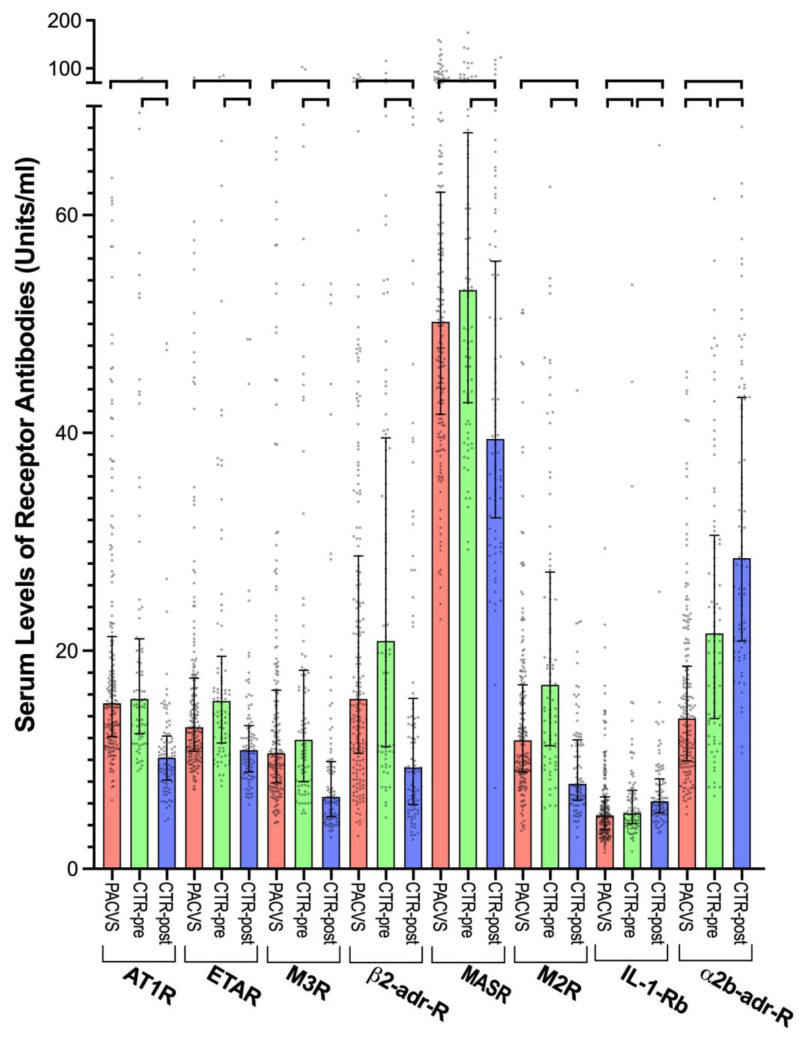
Sera levels of GPCR autoantibodies diverging between control and PACVS samples. Receptor antibodies in the sera of study subjects (n = 191) suffering from PACVS for more than five months after the last vaccination (red, PACVS) and in the sera of healthy volunteers (n = 89) before the first vaccination (green, CTR-pre) and six months after the second vaccination (blue, CTR-post). Vaccination with SARS-CoV-2 mRNA vaccine (Moderna or BioNTech). Dots: single values; boxes and error bars: medians ± interquartile ranges; brackets: significant differences between groups (*p* < 0.0001).

A subset of eight of the analyzed receptor antibodies differed significantly (*p* < 0.0001) between post-vaccination sera (6 months after the last vaccination) in the control cohort and post-vaccination sera (>5 months after the last vaccination) of PACVS-afflicted persons (Figure 2, compare red with blue columns). Six of these antibodies (AT1R, ETAR, M2R, M3R, β2-adr-R, MASR) were significantly (*p* < 0.0001) higher in PACVS subjects than in post-vaccination controls. Coincidentally, these six receptor antibodies exhibited vaccination-associated decreases in controls (Figure 1). Consequently, the serum levels of these antibodies were higher in the PACVS subjects than in post-vaccination controls (Figure 2, compare red with blue columns) but were similar to pre-vaccination controls (Figure 2, compare red and green columns). In contrast, antibodies against IL-1-Rb and α2b-adr-R were significantly (*p* < 0.0001) lower in PACVS subjects than in controls (both pre-and post-vaccination) but exhibited vaccination-associated increases in controls (Figure 1). It should be noted that the above PACVS-associated alterations in circulating receptor antibodies were not associated with any particular vaccination regimen listed in Table S1.

**Figure 3:**
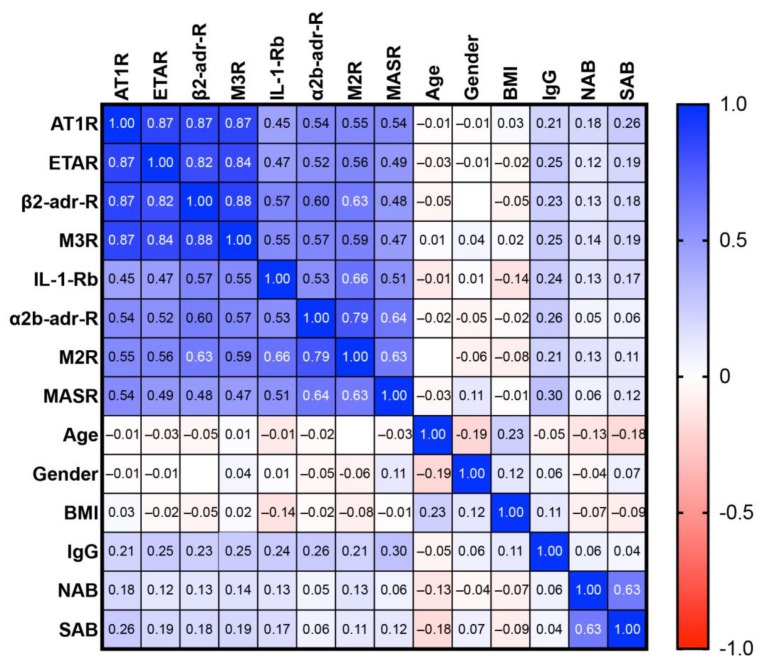
Covariances of receptor antibodies in PACVS (N = 191). Correlative heatmap of serum levels of GPCR antibodies (AT1R, ETAR, b2-adr-R, M3R, IL-1-Rb, a2b-adr-R, M2R, MASR), antibodies against SARS-CoV-2 spike S1 protein (SAB) and nucleocapsid protein (NAB), total IgG (IgG), age, gender and body mass index (BMI). Numerical values: Spearman’s R values ≥ 0.7 are considered significant (*p* < 0.0001).

The 95% confidence intervals of the eight receptor antibodies that were different in PACVS did not overlap between PACVS subjects and post-vaccination controls (Figure 2 and Table 1), suggesting that these receptor antibodies might act as biomarkers allowing serological discrimination of PACVS from the normal post-vaccination state. This assumption was tested by analyzing receiver operator characteristics (ROC). All eight candidate receptor antibodies exhibited significant areas under the ROC curve (Table 2 and Figure S2). The sensitivities for discriminating PACVS subjects from post-vaccination controls at 95% specificity (based on the confidence limits of the post-vaccination controls) ranged from 40 to 90% (Table 2), which indicates that not all the receptor antibodies had similar predictive powers. Moreover, cross-correlation analysis of the above receptor antibodies (Figure 3) revealed two clusters of significant covariance (Spearman’s R ≥ 0.7, *p* < 0.0001), one consisting of AT1R, ETAR, M3R, β2-adr-R and the other consisting of α2b-adr-R and M2R. Of note, PACVS-relevant receptor antibodies were not correlated (Spearman’s R < 0.7, *p* > 0.1) with total IgG, COVID-serology (SAB, NAB), gender, age or body mass index (BMI), excluding these factors as confounders (Figure 3). Optimal discrimination of PACVS subjects from post-vaccination controls was obtained based on increases in AT1R, and MASR and decreases in IL-1-Rb and α2b-adr-R relative to the 95% confidence limits of healthy post-vaccination controls. Under these conditions, AT1R and α2b-adr-R exhibited good sensitivities (90.1 and 89.5%, respectively) and MASR and IL-1-Rb exhibited moderate sensitivities (71.8 and 66.5%, respectively) for PACVS (Table 2).

### 3.3. Discrimination of PACVS from Post-Vaccination Controls Based on Interleukins

**Figure 4:**
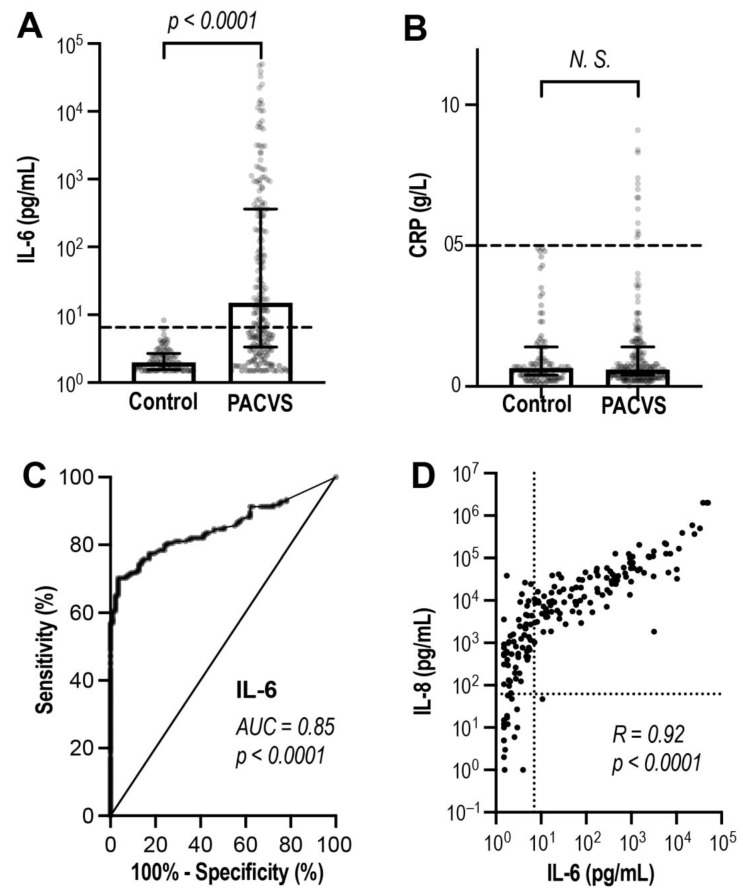
Inflammation markers in post-vaccination controls and PACVS-affected subjects. IL-6 (**A**) and CRP (**B**) were compared in post-vaccination controls (Controls, *N* = 88) and PACVS subjects (PACVS, *N* = 191) using U-test. (**C**) IL-6 values were subjected to ROC analysis (identity indicated by solid line). (**D**) Spearman correlation analysis of IL-6- und IL-8 values from PACVS subjects. Boxes: medians; error bars: interquartile ranges; brackets: results of U-test (N.S.: not significant). Dotted lines: 99% confidence limits of reference values corrected for age and gender in (**C**).

We compared PACVS subjects and post-vaccination controls using a basic panel of potentially relevant laboratory markers encompassing total IgG (IgG), SARS-CoV-2 serology (SAB, NAB), cardiac markers (proBNP, Troponin T) and inflammation markers (IL-6 and C-reactive protein, CRP). Of these parameters, only IL-6 was identified as a potentially discriminative biomarker of PACVS (Table 2 and Figure 4). IL-6 levels increased above the reference level in most PACVS subjects and were significantly (*p* < 0.0001) higher than in post-vaccination controls (Figure 4A). ROC curves indicated reasonable discrimination of PACVS subjects from post-vaccination controls based on IL-6 (AUC = 0.85, Figure 4C). Interestingly, CRP was similar in PACVS subjects and controls (Figure 4B), while in PACVS, the increased levels of IL-6 were linearly correlated with even more pronounced increases in interleukin 8 (IL-8) (Figure 4D). IL-6 and IL-8 were thus identified as additional biomarkers of PACVS.

### 3.4. Exclusion of SARS-CoV-2 Infection/COVID-19 Reconvalescence as Confounder of PACVS

Persons suffering from florid SARS-CoV-2 infections were excluded from the study. However, a subgroup of the included PACVS-afflicted subjects (76/191) exhibited NAB-reactivity. A total of 52 of these subjects reported SARS-CoV-2 infections or COVID-19 re-convalescence in their case histories. The other 24 NAB-positive participants appeared to have acquired SARS-CoV-2 infection without noting. In contrast, post-vaccination controls were selected based on the absence of NAB reactivity and no report of SARS-CoV-2 infection during the monitoring period. To exclude NAB reactivity as a possible confounder of PACVS diagnostic biomarkers, we compared candidate biomarkers of PACVS (listed in Table 2) between NAB-positive (*N* = 76) and NAB-negative (*N* = 115) PACVS subjects. All candidate PACVS biomarkers exhibited slightly higher values in NAB-positive than in NAB-negative PACVS subjects (Table 3, first three columns from the left). Most of these differences were small (median effect size < 10%) and insignificant (*p* > 0.5, U-test). Only AT1R and M3R exhibited more pronounced (median effect sizes 12.8 and 20.2%, respectively) and marginally significant (*p* ≤ 0.05, U-test) increases in NAB-positive compared to NAB-negative PACVS subjects. However, corresponding differences in AT1R and M3R between NAB-negative PACVS subjects and NAB-negative post-vaccination controls were much greater (median effect sizes > 40%) and of higher significance (*p* < 0.0001) (Table 3, compare columns 4 and 5). Thus, we assume that the confounding effect of SARS-CoV-2 infections on PACVS diagnosis is very minor and negligible.

## 4. Discussion

### 4.1. Salient Findings

We present a set of observations that are potentially relevant for the understanding and diagnosis of PACVS, a dysautonomia syndrome associated with, and possibly triggered by, SARS-CoV-2 mRNA vaccination [1]:

- In healthy persons not affected by PACVS, the repertoire of receptor antibodies involved in cardiovascular regulation and immune homeostasis undergoes long-term adjustment following SARS-CoV-2 mRNA vaccination.
- The above adjustment seems blunted, absent or even inversed in persons who present clinical phenotypes of PACVS after SARS-CoV-2 mRNA vaccination.
- PACVS-afflicted persons can be distinguished from individuals subjected to SARS-CoV-2 mRNA vaccination without developing PACVS based on serum levels of IL-6/IL-8 and antibodies against AT1R and α2b-adr-R.

### 4.2. Limitations

- Our study is restricted to SARS-CoV-2 mRNA vaccines, for which we had an appropriate control cohort. Whether our findings apply to chronic sequelae following other types of SARS-CoV-2- vaccinations, or even vaccinations in general, remains to be investigated.
- The clinical PACVS phenotype studied here is based on a long list of symptoms. It is heterogeneous and possibly encompasses more than one clinical entity. Moreover, the selection of studied PACVS cases is biased by the exclusion of 71 applicants with potentially confounding co-morbidities or medications who could nevertheless suffer from PACVS.
- The PACVS cohort was recruited five or more months after vaccination. Matching pre-vaccination sera from these same persons could not be obtained. Consequently, vaccination-associated serological alterations in the PACVS cohort could not be determined intra-individually but had to be judged by comparing with a matched post-vaccination control cohort.
- Receptor antibodies were determined by IgG binding to the native receptors. We and others have previously demonstrated that such antibodies can modulate receptor function in several ways [38]; however, the functional properties of receptor antibodies were not directly assessed in this study.
- Our observation has been limited to a period of 5–6 months after vaccination. We do not know how long the observed effects last beyond this period.

### 4.3. The Physiological Response of Receptor Antibodies to SARS-CoV-2 mRNA Vaccination

In persons not affected by PACVS, only 2 of 16 tested receptor antibody species remained unaltered following SARS-CoV-2 mRNA vaccination, whereas 11 decreased and three increased for a prolonged period. This robust and durable response was prevalent in a healthy cohort; therefore, it probably represents a physiological vaccination response of the receptor antibody repertoire comprising two distinct features:

- Downregulation of a cluster of receptor antibodies targeting the renin–angiotensin–aldosterone system and other components of cardiovascular regulation. Incidentally, some of these receptor antibodies are frequently increased in POTS [20,23,24], ME/CFS [18,22,25], severe COVID-19 [28,29,30,31,32], chronic heart failure [39,40] and allograft rejection [41]. The most distinctive candidate of this cluster is the AT1R antibody.
- Two receptor antibodies were upregulated. One of these, the IL-1-Rb antibody, is thought to play a role in immune homeostasis [35] and to have a protective effect against certain rheumatic diseases [42]. The α2b-adr-R receptor, on the other hand, plays a role in thrombogenesis and its inhibition by small molecule antagonists counteracts platelet aggregation induced by adenosine diphosphate, epinephrine or arachidonic acid in blood samples of healthy individuals [43].

### 4.4. Putative Pathogenic Role of Blunted Receptor Antibody Adaptation in PACVS

Receptor antibody levels in sera of PACVS-affected persons were dissimilar from the post-vaccination state but similar to the pre-vaccination state of persons not suffering from PACVS. Thus, PACVS is potentially associated with a lack or attenuation of the physiological adjustment of the receptor antibody repertoire following SARS-CoV-2 mRNA vaccination. This conclusion could not be corroborated with irrefutable evidence because vaccination-naïve sera could not be obtained post festum from the PACVS-affected persons.

Many of the receptor antibodies downregulated in healthy persons and elevated in PACVS subjects have previously been implicated as disease markers, risk factors, pathogens or even therapy targets in POTS [20,23,24], ME/CFS [18,22,25], severe COVID-19 [28,29,30,31,32], chronic heart failure [39,40], allograft rejection [41], rheumatic diseases [42] and various other syndromes and diseases [44]. It is plausible to assume that vaccination-associated downregulation of these receptor antibodies possibly protects against the above diseases and syndromes, while their lack or attenuation mimics them, at least in part. 

Conversely, the IL-1Rb antibody is upregulated in healthy individuals after vaccination but is decreased in PACVS subjects. It targets a receptor involved in cytokine release [35,42]. Upregulation of this antibody could therefore play a role in the limitation of inflammatory responses to SARS-CoV-2 mRNA vaccination. Its lack could contribute to the persistence of the increase in IL-6, which distinguishes the PACVS cohort from the normal post-vaccination state. Interestingly, PACVS-associated upregulation of IL-6 is correlated to an even more pronounced upregulation of IL-8, which has also been observed in post-COVID-19 ME/CFS [33]. 

Vaccination-associated upregulation of α2b-adr-R antibodies could similarly serve a protective purpose. It is conceivable that these antibodies interfere with the stimulation of the α2b-adr receptor on platelets via adenosine diphosphate, epinephrine or arachidonic acid, thereby neutralizing its prothrombotic function [43]. Along these lines, upregulation of the α2b-adr-R antibody following SARS-CoV-2 vaccination could be a mechanism compensating for the prothrombotic stimulus of the vaccination [4]. The lack of that compensatory mechanism in PACVS would be potentiated by concomitant increases in IL-6 and IL-8 promoting thrombosis via other pathways [45]. Similar protective functions have been described for β-adrenergic receptor antibodies in pediatric cardiomyopathy [46] and allergic asthma [47]. 

In summary, these considerations give rise to the attractive speculation that PACVS could result from the inability to respond to SARS-CoV-2 mRNA vaccination with protective adjustments of the receptor antibody repertoire entailing phenotypic mimicry of syndromes associated with corresponding aberrations of receptor antibodies, e.g., POTS, ME/CFS and certain rheumatic diseases [42] and the loss of protective functions of receptor antibodies. It should be noted that PACVS, as presented by the participants in this study, appears distinct from various acute autoimmune phenomena casuistically reported in the context of SARS-CoV-2 vaccination [5].

### 4.5. The Blood Marker Signature of PACVS

Irrespective of the putative pathogenetic role of receptor antibodies in PACVS, a combination of two index receptor antibodies (AT1R and α2b-adr-R) in conjunction with IL-6 allows discrimination of PACVS from the normal post-vaccination state with a cumulative sensitivity and specificity of up to 90%. However, increases in IL-6 [48], IL-8 [33] and AT1R antibodies [28] have also been observed in long COVID-19 and post-COVID-19 ME/CFS. Thus, further studies will be required to find out whether the suggested blood marker signature similarly distinguishes PACVS from vaccination-unrelated forms of potentially confounding diseases such as long COVID-19 and ME/CFS, which were excluded from this study. It is conceivable that the discriminative power of PACVS diagnostic can be improved by adding further independent blood markers identified in this study, most notably antibodies against MASR and IL-1-Rb.

## 5. Conclusions

The fraction of vaccinated persons suffering from PACVS is unknown. Current estimates assume an incidence of 0.02%, amounting to 40,000 affected persons in Germany alone. These patients are currently not treated appropriately for several reasons: (i) The number of unreported cases is high because diagnostic criteria are not established. It is not even generally accepted that the syndrome exists. (ii) The number of false-positive cases is high because PACVS is similar to various diseases and syndromes unrelated to vaccination. Moreover, sequelae of undetected SARS-CoV-2 infections could be erroneously blamed on SARS-CoV-2 vaccination. Due to these factors, PACVS is currently not/rarely diagnosed in terms of a somatic disease. Instead, PACVS cases tend to be classified as psychosomatic or discarded as irrelevant or imaginary.

Our study may help to improve this unsatisfactory situation in two ways: We provide evidence of PACVS as a somatic disease by linking a clinical phenotype with specific pathognomonic alterations in serological markers; thus, we suggest diagnostic criteria for an objective discrimination of PACVS from the healthy post-vaccination condition. These criteria may not be sufficiently specific to separate PACVS from all confounding diseases or for the diagnosis of PACVS in clinical health care. However, the proposed laboratory diagnostic can act as a stringent rule-out criterion, allowing future PACVS studies to focus on the probable cases.

Great care was taken to exclude possible confounders from this study (Figure S3). However, inclusion as defined by the symptoms listed in Table S2 was less focused. As a consequence, the PACVS phenotype emerging from the present study is heterogeneous and probably encompasses more than one clinical entity. We believe that one objective of future studies should be to draw a clearer and more differentiated clinical picture of PACVS and to use the suggested biomarker signature for patient stratification in a prospective study setting.

## Figures and Tables

**Table 1:** Receptor antibodies before/after vaccination and in PACVS.

	Median ^1^	25% Perc.	75% Perc.	∆ vs. PACVS (*p*, U-Test)
AT1R				
PACVS ^2^ (*n* = 191)	15.2	12.1	21.3	-
Contr. pre ^3^ (*n* = 89)	15.6	12.4	21.1	N.S. ^5^
Contr. post ^4^ (*n* = 89)	10.4	8.1	12.4	<0.0001
ETAR				
PACVS (*n* = 191)	13.5	10.8	18.5	-
Contr. pre (*n* = 99)	15.4	11.6	19.5	N.S.
Contr. post (*n* = 89)	11.0	8.8	13.8	0.0001
IL-1-Rb				
PACVS (*n* = 191)	4.9	3.8	6.9	-
Contr. pre (*n* = 89)	5.1	4.2	7.2	N.S.
Contr. post (*n* = 89)	6.2	5.3	8.2	<0.0001
M3R				
PACVS (*n* = 191)	10.6	7.9	16.4	-
Contr. pre (*n* = 89)	11.9	8.0	18.2	N.S.
Contr. post (*n* = 89)	6.6	4.8	9.2	<0.0001
β2-adr-R				
PACVS (n = 191)	12.8	8.9	16.6	-
Contr. pre (n = 89)	20.9	11.2	39.6	N.S.
Contr. post (*n* = 89)	9.3	5.8	14.4	<0.0001
MASR				
PACVS (*n* = 191)	50.2	41.7	62.1	-
Contr. pre (*n* = 89)	53.1	42.8	67.6	N.S.
Contr. post (*n* = 89)	39.2	31.7	45.7	<0.0001
M2R				
PACVS (*n* = 191)	11.8	8.9	16.9	-
Contr. pre (*n* = 89)	16.9	11.3	27.2	<0.0001
Contr. post (*n* = 89)	7.7	6.2	11.7	<0.0001
α2b-adr-R				
PACVS (*n* = 191)	13.8	9.9	18.6	-
Contr. pre (*n* = 89)	21.6	13.8	30.6	<0.0001
Contr. post (*n* = 89)	27.9	20.9	43.2	<0.0001

^1^ Units/mL, ^2^ exhibiting disease symptoms for six or more months after the last vaccination with mRNA vaccine, ^3^ before the first vaccination with Spikevax (Moderna), ^4^ six months after the second vaccination with Spikevax (Moderna), ^5^ not significant.

**Table 2:** Discrimination of PACVS from post-vaccination controls based on receptor antibodies and IL-6.

	ROC (AUC ± SE)	ROC (*p*)	Cut-off (U/mL) ^1^	Sensitivity (%) ^2^
AT1R	0.824 ± 0.027	<0.0001	≤10.7	89.7
ETAR	0.681 ± 0.035	<0.0001	≤11.5	64.9
M3R	0.741 ± 0.034	<0.0001	≤12.4	40.3
β2-adr-R	0.681 ± 0.036	<0.0001	≤11.6	66.5
α2b-adr-R	0.828 ± 0.025	<0.0001	≥25.2	90.3
M2R	0.703 ± 0.034	<0.0001	≥14.2	64.4
MASR	0.675 ± 0.037	<0.0001	≤44.0	72.3
IL-1-Rb	0.913 ± 0.019	<0.0001	≥5.8	66.5
IL-6	0.850 ± 0.022	<0.0001	≥2.3	82.0

^1^ Derived from limits of 95% confidence intervals of post-vaccination controls, ^2^ At 95 % specificity relative to post-vaccination controls.

**Table 3:** Impact of past SARS-CoV-2 infection on candidate markers of PACVS.

	PACVS ± COVID ^1^		PACVS w/o COVID vs. post-vacc. CTR ^2^	
	Median Effect Size ^3^ (%)	Significance (*p*) ^4^	Median Effect Size ^3^ (%)	Significance (*p*) ^4^
AT1R	**+12.8**	**0.01**	**+43**	**<0.0001**
ETAR	+7.9	0.11		
β2-adr-R	+7.2	0.07		
M3R	**+20.3**	**0.05**	**+44.4**	**<0.0001**
IL-1-Rb	+6.3	0.08		
α2b-adr-R	+4.8	0.50		
M2R	+9.5	0.06		
MASR	+4.1	0.40		
IL-6	−1.3	0.33		

^1^ PanIg reactivity against SARS-CoV-2 nucleocapsid protein (*N* = 115 neg, *N* = 76 pos), ^2^ Six months after the second vaccination, SARS-CoV-2 nucleocapsid protein reactivity excluded (*N* = 89). ^3^ Differences between medians of groups, ^4^ *p*-values of differences between groups (U-test), significant differences are in bold.

## Data Availability

The data presented in this study are available on request from the corresponding author. The data are not publicly available due to privacy concerns by the study participants.

## Supplementary Materials

The following supporting information can be downloaded at: https://www.mdpi.com/article/10.3390/vaccines11111642/s1, Figure S1: Covariance of receptor antibodies in sera of healthy volunteers (n = 89), Figure S2: ROC curves of receptor antibodies discriminating PACVS subjects from post-vaccination controls, Figure S3: Flowcharts of inclusion of study participants and controls, Table S1: Vaccination history of participants, Table S2: Metadata and inclusion/exclusion criteria of controls and PACVS subjects.

## Abbreviations

α1-adr-R-AB	Alpha-1 adrenergic receptor antibody
α2a-adr-R-AB	Alpha-2A adrenergic receptor antibody
α2b-adr-R-AB	Alpha-2B adrenergic receptor antibody
α2c-adr-R-AB	Alpha-2C adrenergic receptor antibody
ACE-II-AB	Angiotensin-converting enzyme 2 antibody
AT1R-AB	Angiotensin II type 1 receptor antibody
β1-adr-R-AB	Beta-1 adrenergic receptor antibody
β2-adr-R-AB	Beta-2 adrenergic receptor antibody
CRP	C-reactive protein
ETAR-AB	Endothelin-1 type A receptor antibody
IL-1-Rb-AB	Interleukin-1 receptor type 2 antibody
IL-6/-8	Interleukin 6/8
M1R-AB	muscarinic acetylcholine receptor M1
M2R-AB	muscarinic acetylcholine receptor M2
M3R-AB	muscarinic acetylcholine receptor M3
M4R-AB	muscarinic acetylcholine receptor M4
M5R-AB	muscarinic acetylcholine receptor M5
MASR-AB	MAS 1 receptor antibody
MCAS	Mast cell activation syndrome
ME/CFS	Myalgic encephalomyelitis/chronic fatigue syndrome
NAB	PanIg reactivity against SARS-CoV-1 nucleocapsid protein
pBNP	pro-brain natriuretic peptide
PEM	Post exertional malaise
POTS	Postural tachycardia syndrome
PACVS	Post-acute COVID-19 vaccination syndrome
ROC	Receiver-operator characteristics
SAB	PanIg reactivity against SARS-CoV-1 spike S1 protein
SFN	Small fiber neuropathy
