# Supplementary material for: Chronic Fatigue and Dysautonomia following COVID-19 Vaccination Is Distinguished from Normal Vaccination Response by Altered Blood Markers
Source: Vaccines (Basel). 2023 Oct 26;11(11):1642. doi: 10.3390/vaccines11111642 (PMC10674626; doi:10.3390/vaccines11111642)
Supplement: Supplementary file 1 [file vaccines-11-01642-s001.zip › vaccines-2609388-supplementary.pdf]

Table S1: Vaccination History of Participants

	N
**mRNA Vaccination preceeding PACVS Onset**	
Moderna (SpikeVax)	32
BioNTech (Comirnatry)	159
**Vaccination Cycles before PACVS Onset**	
1 cycle	47
2 cycles	96
3 cycles	48
**Vaccination Regimen before PACVS Onset**	
1x BioNTech	41
2x BioNTech	72
3x BioNTech	27
1x Moderna	6
2x Moderna	11
3x Moderna	6
Moderna/ BioNTech	1
2x BioNTech/ Moderna	8
2x Moderna/ BionNTech	2
Astra-Zeneca/ BioNTech	7
Astra-Zeneca/ Moderna	1
Astra-Zeneca/ 2x BioNTech	5
Janssen/ BioNTech	3
Janssen/ Moderna	1

Table S2: Meta Data and In-/Exclusion Criteria of Controls and PACVS Subjects

**Controls**	*N* = 89 , Gender: 71 female, 18 male (p<0.001)<br>Age (years, range/mean/median): 21 - 64, 39/49 (p<0.0001)
Inclusion:	Initial dual vaccination with Spikevax (Moderna), spaced six weeks<br>Monitoring period: six months after second vaccination<br>Donation of serum sample 48 h before first and 6 months after second vaccination
Exclusion:	De novo disease symptoms during monitoring period:<br>- Immunological/ rheumatological disorders (myalgia, RBV-reactivation, inflammation, arthritis)<br>- Cardiovascular disorder (pressure crisis, heart insufficiency, arrhythmia, sinus tachycardia, ischemia)<br>- Neurologic disorder (cognitive impairment, peripheral nerve dysfunction, muscular fibrillation or paresis)<br>- Other disease or health condition entailing occupational impairment<br>Increased laboratory markers in post-vaccination serum:<br>- IL-6 > 7 pg/ml, CRP > 0.5 mg/dl, pBNP > 125 pg/ml, TpT > 11 ng/ml, IgG < 700, >1600 mg/dL<br>- SARS-CoV-2 nucleocapsid antibodies indicative of passed SARS-CoV-2 infection
**Participants.**	*N* = 191, Gender: 159 females, 32 males (p<0.001)<br>Age (years, range, mean/median): 18 - 66, 40/39 years (p<0.0001)
Inclusion:	Development of symptoms or disease after SARS-CoV-2 vaccination with mRNA-vaccine (Moderna or Pfizer/BioNTech)<br>Established or suspected diagnosis of syndrome or ≥3 symptoms thereof[1]<br>Start of symptoms ≥ 1 week after first and/or ≤ 4 weeks after last vaccination<br>Persistent symptoms for ≥ 5 months<br>Completion of on-line query<br>Donation of serum sample at ≥ 5 months after onset of symptoms<br><br>Diseases/ syndromes (only included if occurring/diagnosed[1] after SARS-CoV-2 vaccination):<br>- Myalgic Enzephalomyelitis/Chronic Fatigue Syndrome (ME/CFS)<br>- Post-Covid-19/long Covid-19 syndrome in the absence of SARS-CoV-2-infection<br>- Post vaccination syndrome<br>- Postural Tachycardia Syndrome (POTS)<br>- Mastcell activation syndrome (MCAS)<br>- Fibromyalgia/chronic pain syndrome<br>- Peri-/Myocarditis<br>- Thrombosis<br>- Small fiber neuropathy (SFN)<br>- Bell's palsy<br>- Guillain-Barré syndrome (GBS)<br>- Vaccine-induced hyperinflammation<br>- Other adverse vaccination reactions including herpes zoster<br><br>Included symptoms (≥3):<br>- Immunological/ rheumatological disorders (e.g. myalgia, joint pain, swollen/painful lymph nodes)<br>- Cardiovascular disorder (e.g. orthostatic intolerance, arrhythmia, sinus tachycardia, palpitations)<br>- Neurologic disorder (e.g. cognitive impairment, peripheral nerve dysfunction, sensomotor paresis)<br>- Other consensus symptoms of ME/CSF [7]
Exclusion:	Potentially confounding chronic medication pre- and post-vaccination<br>- anti-psychotic, anti-depressive<br>- immunosuppressive, anti-proliferative, anti-inflammatory<br>- anti-biotic<br>- immunomodulative treatment after vaccination<br><br>Potentially confounding health condition existing before vaccination<br>- immunological disease (e.g. Post-COVID, Long-COVID, rheumatic disease<br>- musculoskeletal disease (e.g. ME/CFS, Fibromyalgia, Complex regional pain syndrome)<br>- neurological disease (e.g. Multiple Sclerosis, Neuromyelitis Optica spectrum disorders, GBS, Chronic inflammatory demyelinating polyneuropathy (CIPD), Polyneuropathy other origin than CIPD)<br>- Psychiatric disorder (e.g. Depression, Anxiety disorder, Post-traumatic stress disorder, Obsessive compulsive disorder)<br>- Malignancies, transplantations<br><br>Symptoms following other vaccination (incl. non-mRNA SARS-CoV-2 vaccination)

[1] By a physician in hospital or general practice

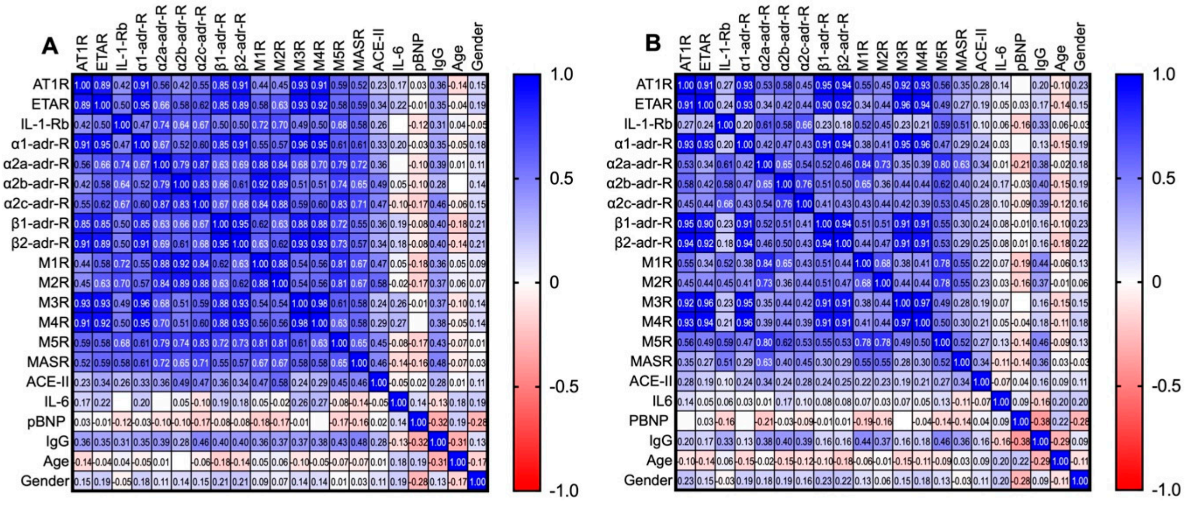

Figure S1. Co-variance of Receptor Antibodies in Serum of healthy Volunteers (n=89): Age, gender and serum values determined 48 h before first (A) and 6 months after second (B) SARS-CoV-2 vaccination (Spikevax, Moderna). Numerical values: Spearmans's R, values ≥ 0.7 considered significant ($p < 0.0001$).

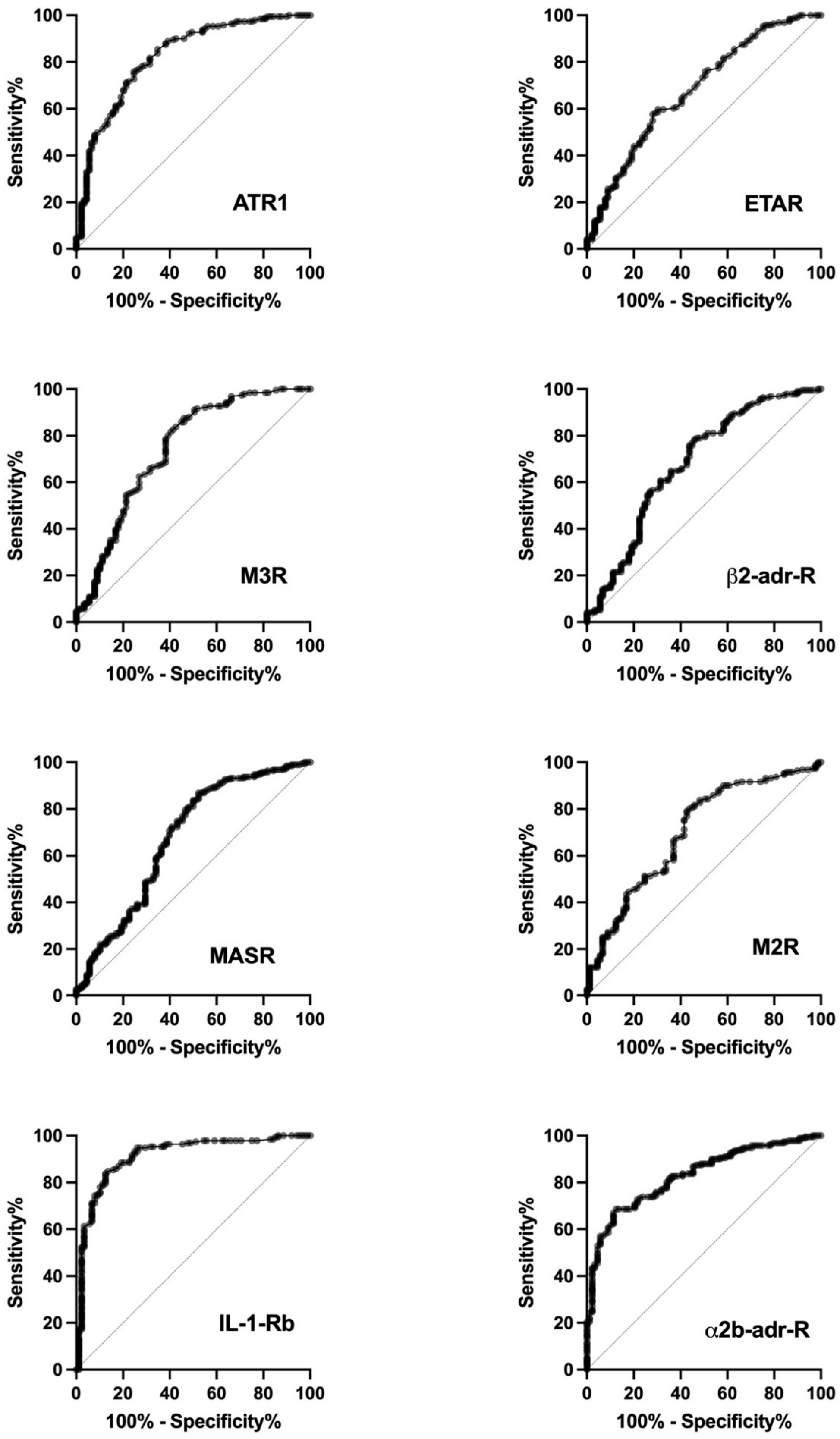

Figure S2. ROC curves of Receptor Antibodies discriminating PACVS Subjects from post-Vaccination Controls

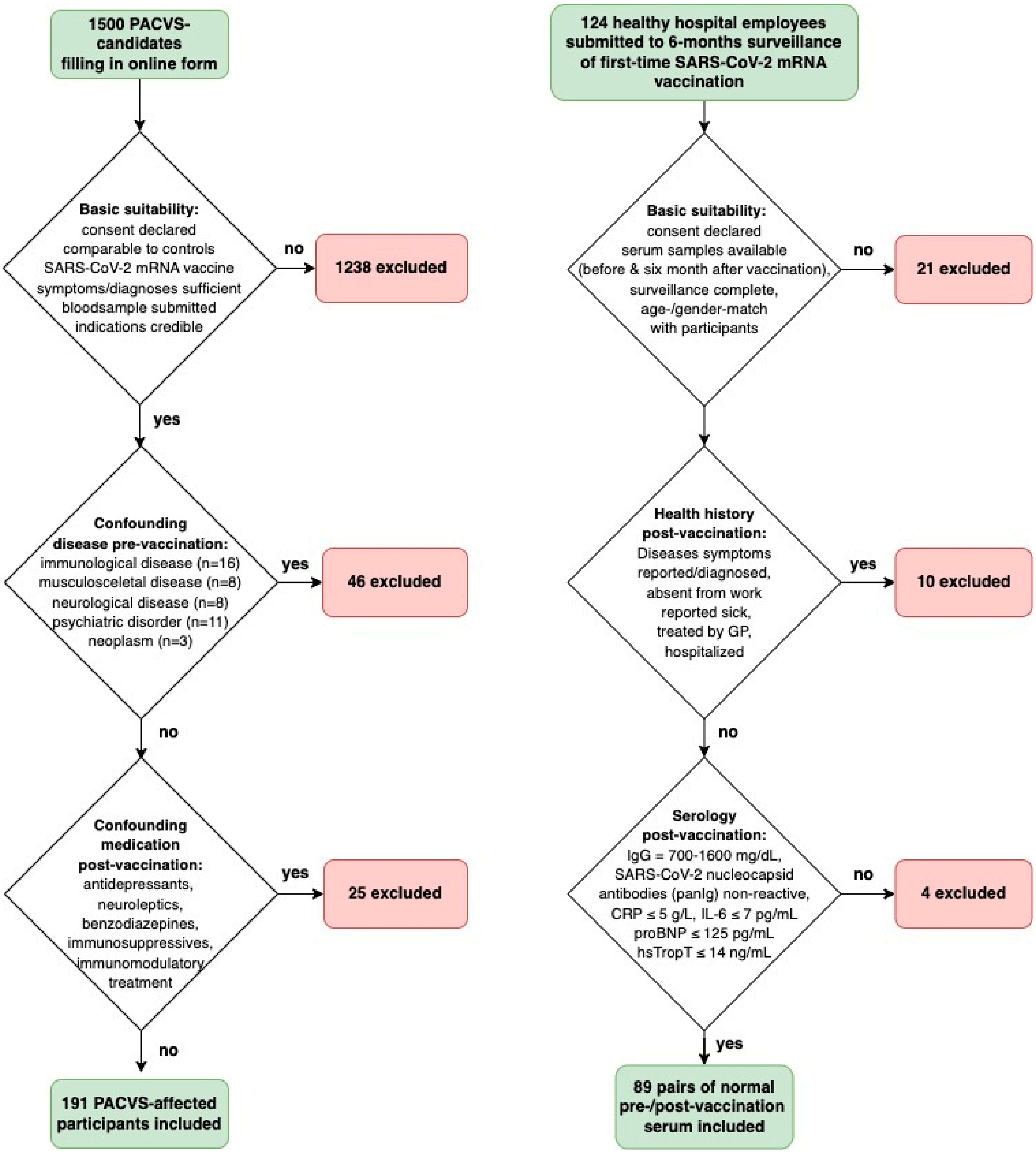

Figure S3. Flow charts of inclusion of study participants (left) and controls (right). In- /exclusion criteria are listed in Table S2 Serological surveillance of healthy hospital employees used as source for controls as described in [37].
